# Switching to Vortioxetine in Patients with Poorly Tolerated Antidepressant-Related Sexual Dysfunction in Clinical Practice: A 3-Month Prospective Real-Life Study

**DOI:** 10.3390/jcm13020546

**Published:** 2024-01-18

**Authors:** Angel L. Montejo, Froilán Sánchez-Sánchez, Rubén De Alarcón, Juan Matías, Benjamin Cortés, Claudia Matos, Tomás Martín-Pinto, Peñitas Ríos, Nerea González-García, José María Acosta

**Affiliations:** 1 Nursing School, University of Salamanca, Av. Donantes de Sangre SN, 37004 Salamanca, Spain; 2 Servicio de Psiquiatría, Hospital Universitario de Salamanca, 37007 Salamanca, Spain; rubenalarcon@saludcastillayleon.es (R.D.A.); jmatiasf@saludcastillayleon.es (J.M.); bcortesm@saludcastillayleon.es (B.C.); clamatos@saludcastillayleon.es (C.M.); tmartinp@saludcastillayleon.es (T.M.-P.); 3 Instituto de Investigación Biomédica de Salamanca (IBSAL), Paseo de San Vicente SN, 37007 Salamanca, Spain; jmacosta@saludcastillayleon.es; 4 Centro de Salud Xàtiva, Xàtiva, 46800 Valencia, Spain; froilansan@hotmail.com; 5 Hospital Universitario Cáceres, 10004 Cáceres, Spain; prios777@hotmail.com; 6 Statistical Department, University of Salamanca, 37004 Salamanca, Spain; nerea_gonzalez_garcia@usal.es

**Keywords:** sexual dysfunction, antidepressant, vortioxetine, depression, sexuality

## Abstract

Treatment-emergent sexual dysfunction (TESD) is one of the most frequent and persistent adverse effects of antidepressant medication. Sexual dysfunction (SD) secondary to SSRIs occurs in >60% of sexually active patients and >80% of healthy volunteers, with this causing treatment discontinuation in >35% of patients. However, this factor is rarely addressed in routine examinations, and only 15–30% of these events are spontaneously reported. A strategy of switching to a different non-serotonergic antidepressant could involve a risk of relapse or clinical worsening due to a lack of serotonergic activity. Vortioxetine appears to have less impact on sexual function due to its multimodal mechanism of action. No studies have been published on the effectiveness of switching to vortioxetine in patients with poorly tolerated long-term antidepressant-related SD in naturalistic settings. Study objectives: To determine the effectiveness of switching to vortioxetine due to SD in a routine clinical practice setting. Methodology: observational pragmatic and naturalistic study to determine the effectiveness of the switch to vortioxetine (mean dosage 13.11 ± 4.03) in 74 patients aged 43.1 ± 12.65 (54% males) at risk of discontinuing treatment due to sexual dysfunction. The PRSexDQ*- SALSEX scale (* Psychotropic-Related Sexual Dysfunction Questionnaire) was applied at two moments: baseline visit and after 3 months of follow-up. Results: global Sexual Dysfunction (SD) measured with the SALSEX scale decreased significantly between the baseline visit (10.32; SD 2.73) and the follow-up visit (3.78; SD 3.68), *p* < 0.001. There was a significant improvement (*p* < 0.001) at the endpoint including decreased libido, delay of orgasm, anorgasmia and arousal difficulties in both sexes. After switching to vortioxetine, 83.81% of patients experienced an improvement in sexual function (43.2% felt greatly improved). Most patients (83.3%) who switched to vortioxetine continued treatment after the follow-up visit. A total of 58.1% of patients showed an improvement in depressive symptoms from the baseline visit. Conclusion: switching to vortioxetine is an effective and reliable strategy to treat patients with poorly tolerated previous antidepressant-related sexual dysfunction in real-life clinical settings.

## 1. Introduction

Antidepressants are frequently associated with sexual dysfunction (SD), particularly SSRI serotonergic agents, dual-action drugs, and clomipramine [1]. Other drugs with different mechanisms of action appear to cause fewer sexual adverse effects (mirtazapine, bupropion, moclobemide). Unfortunately, the real incidence of SD is underestimated, and specific questionnaires must be used. Spontaneous reporting of this adverse effect is around 15–20%, while real figures exceed 60–80% [2,3]. The problem is significant and it is closely associated with treatment dropout, particularly in the case of long-term treatments, and has a negative impact on the quality of life of patients and their partners [4].

Vortioxetine (VOR) seems to have a better profile in terms of SD, although most data comes from clinical trials with registration purposes that may have some methodological limitations, such as the depressive population examined and short-term data [5]. SD rates vary between 0.9% and 45%, depending on the study methodology. Since the serotonergic mechanism of action seems to be closely linked with the etiology and pathogenesis of depression [6], drugs increasing serotonin availability are generally associated with high rates of SD, as this neurotransmitter is very closely involved with inhibition of sexual function, impulsivity, and appetite, among others.

The physiopathogenic mechanisms of these phenomena appear to be multifactorial and complex [7]. One mechanism is the increase in circulating serotonin and the activation of serotonin 5-HT2A receptors, which could affect orgasmic function and sexual interest. Erectile dysfunction appears to be caused by changes in nitric oxide functioning and activation of peripheral adrenergic receptors.

Management of SD has been attempted using various approaches [8]: waiting for spontaneous remission, dose reduction, or switching to another drug with a lower profile of impact on sexual functioning, or use of “antidotes” such as sildenafil or other similar compounds [4,9]. Given the high rates of sexual dysfunction nowadays, which are usually left unaddressed by clinicians, and its impact on patients’ quality of life and treatment discontinuation (estimated at over 35%), this problem must be directly investigated in all patients who receive antidepressants.

In the last 10 years, a very significant increase has been observed in the number of publications addressing this topic, and rising rates of SD have been detected with the use of specific questionnaires [10,11,12], compared to the initial estimates obtained from spontaneous patient reports. Initial data on the incidence of SD obtained retrospectively ranged widely: between 5% and 75% depending on the study methodology used. In previous studies using the validated questionnaire Psychotropic-Related Sexual Dysfunction Questionnaire (PRSexDQ-SALSEX) [10], the mean incidence of SD with SSRIs and dual-action agents was 62.9–80% among sexually active patients [3,13]. Nevertheless, only 14–40% of these patients spontaneously reported any dysfunction in either males or females. Women living with psychiatric illness consider sexuality to be an important part of their quality of life [14].

An American group led by Anita Clayton (University of Virginia) also used a specific questionnaire, the Changes in Sexual Function Questionnaire (CSFQ) [12]. After screening a population sample with inclusion and exclusion criteria, their results were similar to the Spanish series, and contributed data on the low prevalence of bupropion-associated SD, which was shown to be lower than 10% [15]. Surprisingly, reports from medical records of serotonergic antidepressant use obtained through the results of registration clinical trials refer to a very low incidence of SD (2–16%) [16]. These differences in incidence compared to those obtained in real clinical practice are due to the lack of use of specific questionnaires to measure sexual dysfunction and are based on spontaneous communication from patients.

The PRSexDQ-SALSEX questionnaire analyzes the following variables on a scale of severity or frequency: (1) lower libido; (2) delayed orgasm/ejaculation; (3) absent orgasm/ejaculation; (4) erectile/vaginal lubrication dysfunction; and (5) patient’s tolerability of sexual dysfunction and risk of discontinuing treatment. The author and the working group have published numerous studies that use this method to evaluate frequency of SD, risk of discontinuation, impact on quality of life, studies in healthy volunteers, clinical management procedures, and global review studies, such as that in World Psychiatry 2018 [4].

The questionnaire has been translated into multiple languages, including French, English, Italian, German, Portuguese, Greek, Swedish, Finnish, Polish, and Japanese, and has recently also been validated in Mandarin Chinese.

Although SD is a common side effect of all SSRIs and dual-action drugs, the highest rates have been reported with paroxetine for several reasons: its powerful serotonergic action, its effect on increasing prolactin and inhibiting nitric oxide, and its greater anticholinergic effect. The most common problems are reduced desire and delay in achieving orgasm. Erectile dysfunction is less common, although rates associated with paroxetine, citalopram, and venlafaxine at standard therapeutic doses were around 30–40%. Absent orgasm or ejaculation is clearly the most poorly tolerated side effect in patients of both genders.

In contrast, the rates of SD caused by mirtazapine, bupropion, and agomelatine are lower than those of SSRIs [1], due to their different mechanisms of action: mirtazapine blocks postsynaptic 5-HT2 receptors (the stimulation of which has been closely related with the development of ejaculation and orgasm changes); bupropion has a dopaminergic/adrenergic action; and agomelatine is a melatonin receptor agonist and HT2C antagonist.

There appear to be differences between genders. Males over 40 years of age generally tolerate SD worse than females [3], but this is not observed in younger individuals, and at least one third of patients considered discontinuing treatment for this reason. In contrast, other patients, such as those with premature ejaculation, accepted their SD well: the delay in achieving ejaculation experienced after starting antidepressant treatment “normalized” their ejaculatory time. Surveys conducted in large patient series report discontinuation figures between 41.7% and 50.8% [14].

In clinical practice, doctors are often unaware and unable to manage the appearance of these side effects. This approach would avoid the possibility of patients discontinuing treatment, particularly among those who require it in the long term.

With regards to clinical management, the treatment of SD caused by antidepressants has not been examined using controlled and extensive studies. Scant data are available to guide clinicians on the most appropriate choice in each case, and no controlled clinical trials have been performed in this area.

In the ELIXIR study [17] clinicians were asked about their treatment choice in cases of SD due to antidepressants. The results indicated that most psychiatrists opted for no intervention, and preferred to wait for spontaneous remission, and a small percentage chose to switch treatment or to add an antidote. In Spain, [3] a clinical study with more than 2000 patients found a rate of SD of over 80% in patients who were receiving SSRIs or dual-action agents. Spanish doctors opted to wait for spontaneous remission in 25% of cases or switched to bupropion or agomelatine in 30% of cases. Results were better for agomelatine (80% reduction in SALSEX scores) [18,19]. The use of PDE5 inhibitors such as sildenafil, or weekend drug holidays, was very rare.

The main objective of this study is to analyze whether switching to another antidepressant with a different mechanism of action is useful in clinical practice, reducing the frequency of sexual dysfunction and maintaining antidepressant efficacy after switching. The experience in our country shows that while patients with at least 3 months following can benefit from switching to other non-serotonergic antidepressants in order to improve SD, they can be at risk of clinical deterioration or depressive relapses in one in three cases. Therefore, new therapeutic alternatives must be found. In the absence of meta-analyses and specifically designed clinical trials, the recommendations obtained from analyzed data from published studies suggest different levels of evidence including switching to another antidepressant (agomelatine, bupropion or mirtazapine), weekend drug holidays (useful in the absence of orgasm), and PD_5_ Inhibitors, among others.

Due to the lack of an effective treatment with favorable, persistent results in antidepressant treatment-emergent SD, newer products with different mechanisms of action that could have less effect on sexual functioning must be explored. One of them is vortioxetine, which has a multimodal mechanism of action on different receptors, with full agonist effect on 5-HT1A, partial antagonism on 5-HT1B, and antagonist effects on 5HT1D, 5-HT3 and 5-HT7, in addition to displaying dopaminergic, adrenergic, histaminergic, and cholinergic effects. Given the lack of current evidence regarding this topic, specific studies in routine clinical practice and in carefully selected populations are required to confirm these preliminary data.

## 2. Study Rationale

Vortioxetine is a recently developed antidepressant with a novel mechanism of action. Data from clinical trials for registration purposes suggest a neutral, or even beneficial, effect on sexual functioning in depressive patients receiving vortioxetine [20,21], which has since been proved again in a recent phase IV randomized study [22]. A switching study showed that vortioxetine is a safe and effective switch therapy for treating SSRI-induced sexual dysfunction in adults with well-treated MDD [23]. Also, improvement in sexual dysfunction with vortioxetine or escitalopram may be influenced by prior SSRI usage, sex, age (≤45 years, women), and history of one to three major depressive episodes [24]. For example, a recent study in postmenopausal transition women observed less antidepressant-induced SD with vortioxetine when compared to paroxetine [25], although the exact dose was not specified. However, overall limited data have been published to date regarding the effects of this antidepressant on sexual functioning.

Vortioxetine, with this novel mechanism of action, could have some implications for less sexual dysfunction. In a recent randomized, double-blind trial with vortioxetine (15–20 mg/day), treatment-emergent sexual dysfunction symptoms were not significantly different versus placebo using the ASEX Scale [26]. In an open-label, flexible-dose (2.5–10 mg/day), 52-week extension study that evaluated the long-term safety and tolerability of vortioxetine, the rate of adverse events related to sexual dysfunction was low [27]. Moreover, a recent prospective epidemiological study shows that females (but not males) treated with vortioxetine presented better sexual function than those treated with SSRIs or Duals and a lower risk of sexual dysfunction [28].

In a recent review [29], authors stated that vortioxetine is well tolerated, but is associated with significantly increased sexual dysfunction at a dosage of 20 mg; however, vortioxetine was shown to improve previous-treatment-emergent sexual dysfunction in patients with well-treated MDD to a greater degree than escitalopram. These studies show some limitations when studying this topic, such as lack of a control group with sexually active patients in a naturalistic setting, so further specific studies are needed.

Therefore, the aim of this study is to determine the frequency and intensity of sexual dysfunction (SD) after switching to vortioxetine from another antidepressant due to TESD.

## 3. Study Objectives

### 3.1. Primary Objective

- To analyze the effectiveness of the antidepressant switch strategy to vortioxetine for the improvement of sexual dysfunction (measured as total SALSEX score) after 3 months of follow-up in patients with poor tolerance or risk of treatment discontinuation (the risk of discontinuation is defined as a score ≥ 2 in item 5 of the SALSEX).

### 3.2. Secondary Objectives

□ To study the individual tolerance and risk of treatment discontinuation using the PSRSexDQ-SALSEX questionnaire at baseline and to the endpoint.
□ To determine differences in SD between males and females at baseline and to the endpoint.
□ To determine differences in SD between different age groups at baseline and to the endpoint.
□ To determine differences in SD between different levels of severity of depression at baseline and to the endpoint.
□ To determine differences in SD between different dosages of vortioxetine (10–20 mg).

## 4. Methodology

### 4.1. Design

This is a naturalistic, prospective, pragmatic, open-label, one-group study design in a routine clinical practice setting, measuring the outcomes of antidepressant switching to vortioxetine in patients with previous antidepressant-related poorly tolerated SD.

### 4.2. Study Subjects

#### 4.2.1. Sample Size Calculation

To analyze the effectiveness of the antidepressant switch strategy to vortioxetine for the improvement of sexual dysfunction, and considering the frequency of SD of different antidepressants in previous studies, the sample size necessary for the difference between proportions was calculated. Assuming a 95% confidence level and a power of 80%, according to data from previous studies in which the proportion of patients with SD on SSRIs was 70% and 45% on dual-action drugs [2,3], and based on recent studies with the same design, the required sample size is 124 patients, with a sample size of 62 male and 62 female patients in each group in order to observe possible gender differences.

#### 4.2.2. Inclusion Criteria

□ Patients who showed at least moderate intensity in their total SALSEX score, with a score ≥ 6 (including ≥2 in item 5, tolerance of sexual dysfunction).
□ Patients with normal sexual function prior to taking antidepressants (normal sexual function was defined as an absence of habitual dysfunctions of sufficient intensity to cause subjective discomfort in the patient in the areas of desire, orgasm or sexual arousal that would require specialized attention, with previous regular, satisfactory sexual and/or autoerotic practices).
□ Sexually active patients treated with an antidepressant for at least 2 months. This time requirement was included to avoid false negatives, as some symptoms do not appear until after this period (loss of sexual desire or erectile/vaginal lubrication dysfunction).
□ Previous antidepressant-related sexual dysfunction. Patients were switched to vortioxetine only if there were symptoms of sexual dysfunction that were considered associated with the previous antidepressant.
□ Treatment exclusively with antidepressants used within approved label (including SSRIs, SNRIs). Combined treatment with benzodiazepines at low clinical doses was permitted (less than 20 mg clorazepate or equivalent).
□ Patients with at least partial response with a maximum score on the Clinical Global Impression Scale of Depression (CGI-D) ≤ 3-mild depression.

#### 4.2.3. Exclusion Criteria

□ SD prior to starting administration of the antidepressant. (Only a mild decrease in libido before starting antidepressant treatment was permitted, as this is considered a symptom of depression itself, although worsening of libido because of treatment was considered as an adverse effect.)
□ Combination of the antidepressant with antipsychotic drugs or mood stabilizers.
□ Use of hormones or any other medication with known capacity to interfere in sexual relationships (antiepileptic drugs, H2 antagonists, recent introduction of contraceptives as concomitant therapy, β-blockers, opiates, and antihypertensive drugs).
□ Medically significant intercurrent diseases clearly affecting sexual function.

#### 4.2.4. Switching Procedures

Switching from previous antidepressant therapy (SSRI, SNRI) was undertaken with no abrupt interruptions. Doses were increased up to 10 mg/day during the first week and, following a naturalistic design, an increase up to 15–20 mg/day of vortioxetine was allowed after the first week. Previous antidepressants were simultaneously gradually tapered down by halving the dose for the first week before complete withdrawal.

#### 4.2.5. Sites

All patients were attended by psychiatrists working in Salamanca’s (Spain) complete outpatient network, which comprises five outpatient units in total.

### 4.3. Variables

#### 4.3.1. Primary Variable

□ The Severity of global SD was measured using the SALSEX total score (scoring from 0 = no sexual dysfunction to 15 = maximum sexual dysfunction) at baseline and to the endpoint. The severity of each individual dimension of sexual functioning (reduced sexual desire, delay of orgasm, anorgasmia, and arousal difficulties such as erectile dysfunction or vaginal lubrication) was measured with a Likert scale (0 = no SD; 3 = maximum SD) of the items 1–4 of the SALSEX questionnaire at baseline and at endpoint visit.

#### 4.3.2. Secondary Variables

□ To study the individual acceptance of SD and risk of treatment discontinuation, we used the score of item 5 of the SALSEX questionnaire (0 = no risk of discontinuation; 3 = maximum risk) at baseline and at endpoint visit.
□ To determine differences in SD that varied across the severity of depression we used the Clinical Global Impression-Improvement (CGI-I) scale for depression, and the CGI-S for sexual functioning at baseline and at endpoint visit.

### 4.4. Data Collection and Analysis

#### 4.4.1. Measurement Scales

##### PRSexDQ-SALSEX Questionnaire

The SALSEX questionnaire for the Evaluation of Psychotropic-Related Sexual Dysfunction was used to measure and evaluate the prevalence and severity of SD (validated in 2008 by Montejo et al. [10] in a population with depressive disorders). This questionnaire was administered at baseline and during the follow-up period (within 3 months of the initial visit). It is included in an annex at the end of this paper. The SALSEX questionnaire is meant to be administered during a direct clinical interview to collect information on whether treatment-related SD is detected, and if it is present, to note whether the patient reported the SD spontaneously or not. The degree of SD is evaluated according to 4 items, for each of the possible manifestations of SD: (1) sexual desire; (2) delayed orgasm; (3) absent orgasm; (4) erection-lubrication. A fifth item evaluates the acceptance of SD, if present. Each of these items is scored between 0 (no problem) and 3 (maximum intolerance).

The presence and severity of SD is evaluated according to the total score and individual item scores of the questionnaire, using the following criteria:

○ No SD: Total score of 0 or 1, only if item 1 is evaluated as a slight loss of libido (which is equivalent to a score of 1 on that item).
○ SD present: Total score of 2–15, or total score of 1 if any item except item 1 (desire) is scored 1.
  □ Mild SD: 1–5 points, provided no item scores ≥2 points and item 5 (tolerability) is not >1.
  □ Moderate SD: 6–10 points, provided that no item scores ≥3 points, or <6 points if any item = 2 and provided that item 5 (tolerability) is not >2.
  □ Severe SD: 11–15 or <11 points if any item = 3 or whenever item 5 (tolerability) = 3.

Clinical Global Impression Scale, severity subscale, applied to sexual dysfunction (CGI-S-SD) to assess the DS severity at baseline
Clinical Global Impression Scale, improvement subscale, applied to sexual dysfunction (CGI-I-SD) to assess the clinical effectiveness of the intervention, administered in the follow-up visit (performed within 3 months of the baseline visit)
Clinical Global Impression Scale, severity subscale (CGI-S), applied to the psychiatric disease for which the antidepressant treatment is administered, to assess severity at baseline
Clinical Global Impression Scale, improvement subscale (CGI-I), to assess the clinical effectiveness of the intervention, administered in the follow-up visit (performed within 3 months of the baseline visit)

##### Adverse Events Assessments

Adverse events (including pre-treatment adverse events) had to be recorded on an Adverse Event Form. The investigator had to provide information on the adverse event, preferably with a diagnosis, or at least with signs and symptoms; start and stop dates (and start and stop time if the adverse event lasted less than 24 h); severity; causal relationship to the IMP; action taken; and outcome. If the adverse event was not related to the IMP, an alternative etiology had to be recorded, if available. If the adverse event was an overdose, the nature of the overdose had to be stated (for example, medication error, accidental overdose, or intentional overdose). If the adverse event was serious, this had to be indicated on the Adverse Event Form. The sponsor/investigator had to comply with all national rules and regulations concerning the reporting of Serious Adverse Events as defined in the ICH GCP guidelines and forward a copy of any such reports to Lundbeck, and to report all AE/ADRs to Lundbeck immediately. ICH.

#### 4.4.2. Visits

Information was collected on two visits:

□ Baseline visit (V1): treatment initiation switch if prompted by poorly tolerated SD;
□ Visit 2 (V2) conducted within 3 months of follow-up after switching antidepressant at baseline visit.

#### 4.4.3. Treatment

Patients switched treatments only if the clinician and/or the patient considered that some treatment modification was necessary to improve SD due to the use of the current antidepressant, which was replaced by vortioxetine with the patient’s consent.

#### 4.4.4. Ethical and Legal Considerations

##### Patient Information

Informed consent was obtained from the patient, and the physician involved the patient and his/her partner in the study, since their collaboration was deemed essential.

##### Confidentiality

Collection, processing, and transfer of study data were conducted in compliance with the provisions of the Spanish Organic Law 15/1999 on Personal Data Protection. All information on the identity of participating patients was treated as confidential for all purposes. The identity of the patients was not to be disclosed or shared, except when necessary for their treatment, evaluation, follow-up, and safety. The patient was identified in the data collection forms with a patient number. The collected data were entered in a database following a procedure that ensured total dissociation between these data and the identity of the patient.

## 5. Data Analysis

### 5.1. Evaluation Criteria and Data Management

Case report forms (CRFs) were individually reviewed to ensure that all data had been collected or otherwise a reason had been provided. Numerical values were assigned to the open text fields, particularly for adverse reactions. Incorrect and incomplete CRFs, and those that had not been completed according to the protocol were rejected or returned to the corresponding investigator for review and correction.

CRF data considered valid was entered in a database created for this purpose with appropriate safety measures and internal coherence rules, after which any cases with anomalous or inconsistent values were reviewed.

### 5.2. Evaluation Criteria

Cases rejected due to serious inconsistencies or incorrect or incomplete data were not evaluated. All patients were to be described in the participant’s biodemographic characteristics section, indicating the total number of patients included, the total number of patients excluded or who had discontinued treatment early (along with the reason), and the total number of evaluable patients. Pharmacovigilance assessment was based on all recruited patients, except for those cases rejected due to incorrect data or those who did not return to any visit after baseline.

#### Evaluations Performed

General characteristics of patients included in the study, including biodemographic data and their psychiatric diagnosis, were described. The primary analysis was con-ducted by describing the percentage of patients who had SD during previous treatment and after switching to vortioxetine with their respective confidence intervals. Total SALSEX score, frequency, and intensity of each item of the SALSEX (items 1–5) was compared at endpoint vs. baseline.

Testing of the hypothesis was considered significant when the corresponding *p*-value was less than 5% (α = 0.05) for two-tailed tests. All operations were performed on PC-type compatible computers, protected by strict measures controlling access and quality, using the SPSS package, version 23.0 for Windows or a subsequent version.

Measures of central tendency and dispersion for quantitative variables were de-termined throughout the study. Normality of distribution of continuous variables was tested using the Kolmogorov-Smirnov test for one sample, so that the data were sub-sequently analyzed based on the results obtained. The descriptive analysis included categorical qualitative variables measured using frequencies and percentages.

For the inferential statistical analysis, testing of the hypothesis was evaluated using the appropriate parametric test for variables with normal distribution, or non-parametric tests for those with non-normal distribution. Throughout the study, it was of interest to examine differences between the patient groups in the characteristics evaluated. When experimental data were measured using continuous scales that follow a normal distri-bution, the appropriate procedure was the Student’s *t* test for two independent samples. If these scales did not have a normal distribution, a non-parametric Mann–Whitney *U* test was used. If the difference of means was to be studied between more than two groups, the corresponding parametric or non-parametric test was used.

Pearson’s chi-squared test for independence and Fisher’s exact test were used to compare independence between categorical variables.

## 6. Project Development Stages

The study was conducted in the Department of Psychiatry of the Hospital Universitario de Salamanca, with the participation of 10 investigators from 5 mental health outpatient units located in Salamanca, which provides care for a population close to 300,000 inhabitants.

The study was performed in three phases between July 2019 and September 2022. Recruitment was delayed due to COVID-19.

All administrative permissions, including submission of the protocol for qualification by the Spanish Agency of Medicines, agreement from the Research Ethics Committee of the Salamanca Health Area, and agreement from the Government of Castile and Leon were obtained in 2019. An investigators’ meeting was held in January 2020 for an explanation of the protocol, standardization of procedures, training, and practical administration of the SALSEX questionnaire to reduce inter-investigator variability. The collection of baseline sociodemographic data was obtained and included in the CRF, as well as all selection criteria, presence and severity of the psychiatric disease (measured using the CGI-S scale), presence and severity of SD (measured with the SALSEX questionnaire and the CGI-S-SD), and randomized therapeutic strategy of antidepressant switch for the management of SD associated with antidepressant treatment.

Clinical follow-up of patients with SD detected at the baseline visit included the measurement, within 3 months of baseline, of the effectiveness of the intervention using the SALSEX scale (lower scores indicate improvement), and a Clinical Global Impression-Improvement for depression (CGI-IDep) and for sexual functioning (CGI-ISex) after intervention. A determination of the number of patients withdrawing from the study due to lack of efficacy, adverse effects or loss to follow-up was performed.

## 7. Results

103 patients treated with an antidepressant who had poorly tolerated sexual dysfunction after initiation of treatment and who met the inclusion and exclusion criteria of the protocol were switched to vortioxetine. The response to the SALSEX scale was analyzed in 74 patients from whom data were obtained at the follow-up visit (3 months after the baseline). 29 out of 103 patients (28.15%) were lost to the study with no data at Visit 2. Clinical and sociodemographic characteristics of patients switched to vortioxetine are showed in Table 1.

### 7.1. Previous Antidepressant Treatment at Baseline

**Figure 1:**
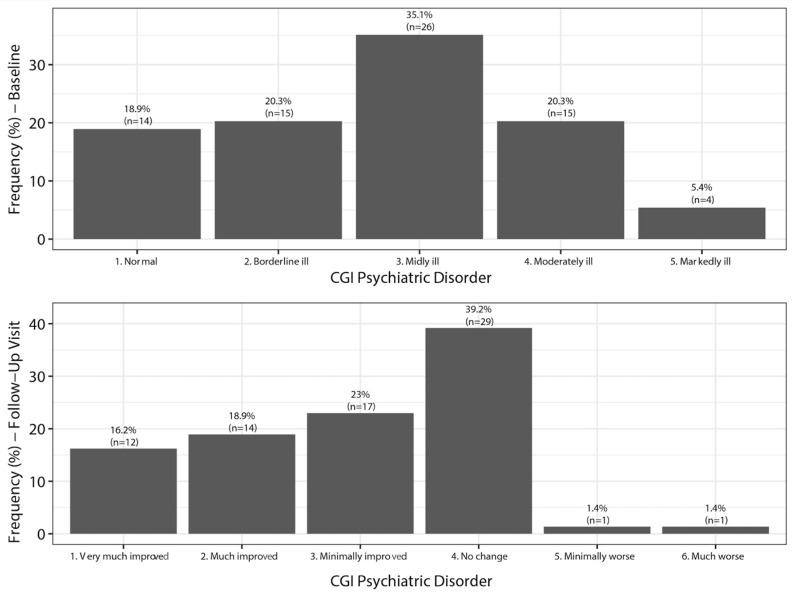
Changes in the Clinical Global Impression of severity of depression (CGI-D). Baseline and follow-up.

At baseline, patients with sexual dysfunction were taking venlafaxine 6.8%, mean dosage 177 mg/day ± 73.02; escitalopram 25.7%, 14.74 mg/day ± 3.90; citalopram 5.4%, 23.75 mg/day ± 7.50; paroxetine 9.5%, 22.86 mg/day ± 7.56; sertraline 18.9%, 78.57 mg/day ± 37.80; fluoxetine 8%, 23.33 mg/day ± 8.17; duloxetine 10.8%, 67.50 mg/day ± 21.21; clomipramine 4.1%, 75.00 mg/day ± 0.000 and desvenlafaxine 10.8%, 78.57 mg/day ± 56.70 (Figure 1).

### 7.2. SALSEX Scale Total Score of Sexual Dysfunction

The total score of Sexual Dysfunction (SD) measured with the SALSEX scale (ranging from 0 = no sexual dysfunction to 15 = strong SD) at the baseline visit in which patients were taking an antidepressant was 10.32 (SD 2.73). The total score on the SALSEX scale in the follow-up visit (3 months after switching to vortioxetine) was 3.78 (SD 3.68). Highly significant differences were found between the global SALSEX score at baseline and after switching to vortioxetine (t = 12.279, *p* < 0.001).

At baseline, 4% of patients had mild sexual dysfunction (SD) (*n* = 3), 20.3% of patients had moderate SD (*n* = 15) and 75.7% of patients had severe SD (*n* = 56).

SD was spontaneously reported in 47.3% of patients (*n* = 35), compared to 52.7% (*n* = 39) for whom it was not.

Significant differences were found in the total score on the SALSEX scale between males and females, with females being higher at baseline (*p* = 0.021), and also after follow-up (*p* = 0.021) (Table 2). There were no differences in age, treatment time to baseline, treatment time to visit 2, or dose.

### 7.3. Clinical Global Impression of Severity of Depression (CGI-D)

Based on analysis of the CGI-D at baseline, 81.1% of the patients switched to vortioxetine felt depressed (35.1% mildly ill, 20.3% borderline, 20.3% moderately ill, and 5.4% markedly ill). After treatment with vortioxetine, 39.2% felt no change, but 58.1% of patients showed an improvement (Figure 1).

### 7.4. Clinical Global Impression of Severity of Sexual Dysfunction (CGI-SEx)

**Figure 2:**
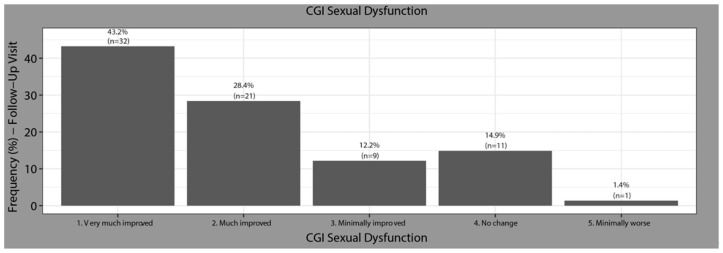
Changes in the Clinical Global Impression of severity of sexual Dysfunction (CGI-Sex). Baseline to follow-up.

Based on analysis of the CGI-Sex at baseline, 100% of patients had sexual dysfunction (6.9% borderline ill, 6.8% mildly ill, 43.8% moderately ill, 28.8% markedly ill, 9.6% severely ill, and 4.1% extremely sick). After switching to Vortioxetine, 83.81% of patients felt an improvement (43.2% felt greatly improved) (Figure 2).

After switching to vortioxetine, 18.9% did not show SD (*n* = 14), 44.6% had mild SD (*n* = 33), 14.9% moderate (*n* = 11), and 14.9% severe (*n* = 11). Five patients (6.7%) did not respond to item 5 of tolerability and that is why they were not classified in any group of the scale, although they presented values of 0 points in the rest of the items.

### 7.5. SALSEX Scale by Items from Baseline to Follow-Up

Analyzing the items of SALSEX at baseline, 85% of patients showed moderate/severe decreases in sexual desire, 86.5% showed moderate/intense orgasm delay, 51.4% showed anorgasmia frequently and 17.6% always/almost always. A similar issue happened with arousal difficulties (Table 3).

At the follow-up visit after switching to vortioxetine, 43.2% of patients no longer showed any problems with sexual desire and 36.5% showed a mild decrease. A similar improvement in orgasm delay, anorgasmia and arousal dysfunction was observed at follow-up (Table 3).

At the follow-up visit, a general tendency to decrease the severity of sexual dysfunction from moderate/severe to becoming mild or absent in all items of the SALSEX was observed in all items (Table 4).

**Figure 3:**
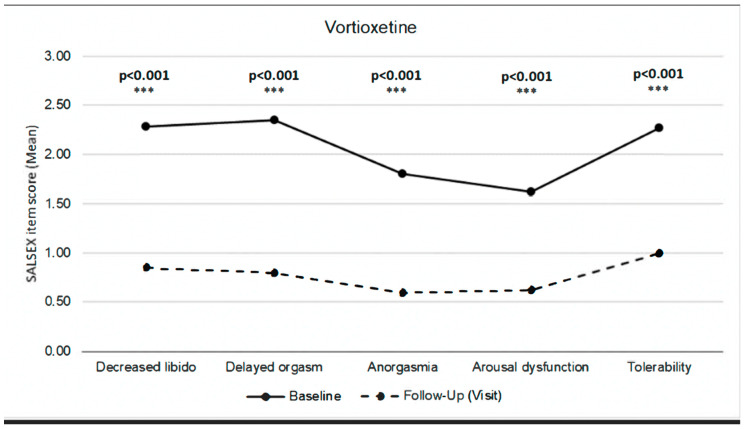
SALSEX score by items. Comparison between baseline and follow-up.

After switching to vortioxetine, a clinically significant improvement (*p* < 0.001) was observed in sexual desire, delayed orgasm, anorgasmia, and arousal difficulties. Additionally, tolerability of sexual dysfunction improved significantly in patients who maintained some degree of sexual dysfunction at the follow-up visit (Figure 3).

### 7.6. Sexual Dysfunction and Dosages of Vortioxetine

**Figure 4:**
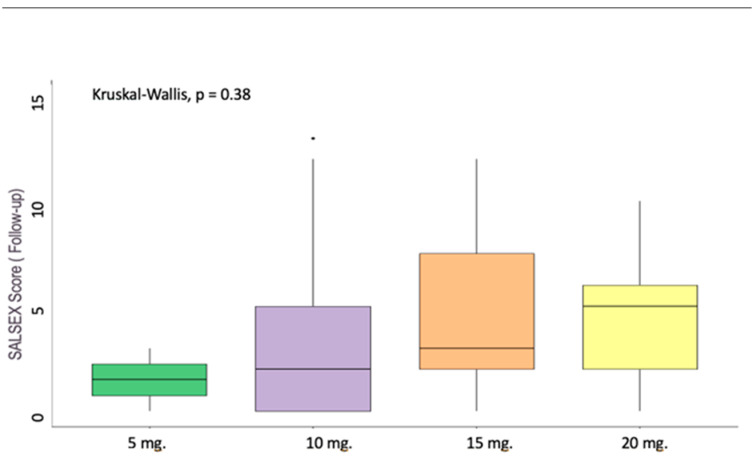
SALSEX score and dosages of Vortioxetine. Vortioxetine 5 mg *n* = 2 (2.70%); 10 mg *n* = 37 (50.00%); 15 mg *n* = 22 (29.73%); 20 mg *n* = 13 (17.57%).

Regarding differences in the total SALSEX score at the follow-up for patients switched to vortioxetine, no significant differences were found comparing the dose administered to patients, although it could be observed at the descriptive level that there was an increase in the SALSEX score as the dose increased (Figure 4).

### 7.7. Clinical Outcomes after Switching to Vortioxetine

Of those switched to vortioxetine (*n* = 103), 70.7% of patients continued treatment (*n* = 65) after three months, two patients voluntarily abandoned treatment due to lack of efficacy, and the investigator decided to withdraw the treatment due to lack of efficacy in two patients. Five patients voluntarily abandoned treatment due to adverse effects, and in two patients the investigator withdrew the treatment due to one or more side effects. Fourteen patients (15.2%) received were not evaluable due to lack of sexual activity. There is no information on the outcome of the strategy for 13 patients. Finally, 65/74 patients switched to vortioxetine continued treatment after the follow-up visit (83.3%).

## 8. Discussion

### 8.1. Value of the Study

Treatment-emergent sexual dysfunction (SD) is one of the most frequent and persistent adverse effects of antidepressant medication and switching to other non-serotonergic antidepressants seems to be the best possible strategy in real clinical practice when SD is poorly tolerated. Some antidepressants appear to have less impact on sexual function due to their reduced serotonergic effect (agomelatine, bupropion, and desvenlafaxine) [30,31,32] or their multimodal mechanism of action like vilazodone [33].

Such is the case for vortioxetine, a recently developed antidepressant with a novel and promising receptor-binding profile [34]. Data from clinical trials for registration purposes suggest a neutral, or even beneficial, effect on sexual functioning in depressive patients receiving vortioxetine [20,21,35]. However, data from clinical trials are characteristically unreliable when it comes to determining SD incidence, as they are derived from biased samples, sexually inactive patients, or short treatment periods. Limited data have been published to date on the effects of this antidepressant on sexual functioning in routine clinical practice.

In our study, we wanted to find out if switching to vortioxetine proved to be an effective strategy when dealing with antidepressant-induced SD. There is to our knowledge only one very recent observational study in a naturalistic setting that explored switching to other antidepressants with a non-serotoninergic mechanism of action as a measure for improving antidepressant-induced SD, and that included vortioxetine as a possible option [36]. This Italian retrospective cohort study tried to analyze several possible solutions for antidepressant-induced SD and, among several options, found vortioxetine to be the most common and effective one, particularly at 10 mg. Although the sample was small (*n* = 30) and was limited to a male population, these results match our own: vortioxetine improved SD consistently when compared to our patients’ previous antidepressants, with 83.81% of patients feeling improved (43.2% feeling greatly improved).

This becomes clearer if we look at other studies with non-serotoninergic antidepressants like agomelatine. In a study comparing agomelatine to venlafaxine as a first prescribed drug to depressed patients [32], agomelatine demonstrated a lower SD incidence rate but overall differences did not exceed 8% of patients.

Regarding our secondary objectives, in our sample, we had a higher rate of SD in females than in males both at baseline and at follow-up, but no differences at all were found in SD among age groups. A study by Jacobsen saw greater improvement of SD in women under the age of 45 with vortioxetine [24] but our results did not match these findings.

We should also address that switching to vortioxetine did not compromise antidepressive efficacy at all either: the CGI-S for depression did not change from baseline to follow-up, and even improved in some cases.

The degree of SD improvement with vortioxetine seems to be highly dependent on dosage: lower doses like 10 mg are more beneficial for SD than higher ones like 15 or 20 mg across several studies [24,36,37,38]. Numerically, our study correlated higher SALSEX scores to higher doses (but even then, lower than with the previous drug), although this was not statistically significant probably due to a lower sample number among dosing subgroups than we originally intended.

Previous studies have already shown vortioxetine to have a minor rate and intensity of SD when compared to SSRI. For example, a double-blind clinical trial was conducted in healthy volunteers comparing sexual dysfunction associated with the use of paroxetine (20 mg/day), versus vortioxetine 10 and 20 mg/day and placebo for 5 weeks [22], finding that the 10 mg vortioxetine dose caused less SD than paroxetine. This difference was also somewhat present at the 20 mg vortioxetine dose, but it did not reach statistical significance due to apparent subject noncompliance. Overall, these results are in concordance with previous comparisons undertaken in depressed patients with vortioxetine and escitalopram [23], confirming and even improving on the previously reported SD incidence rates in the vortioxetine drug label analysis [39]. Additionally, a recent retrospective real-world study has shown that vortioxetine improves sexual functioning in depressed patients after 12 weeks [40].

However, these studies used the CSFQ-14 and did not compare the impact of vortioxetine across several sexual dimensions to other antidepressants. A study on a Spanish population did [28], albeit with a different outcome measure than in this study. Our findings, measured through the PRSexDQ-SALSEX scale, point to vortioxetine having the potential to improve all the previously affected sexual dimensions, but improving decreased libido (in some cases, even restoring it completely) and delayed orgasm slightly more than anorgasmia and arousal dysfunction.

All patients took the previous antidepressant for more than 14 months and most had responded completely to treatment or were mildly ill. Only 20.3% felt moderately ill and 5.4% markedly ill, according to the Clinical Global Improvement of Depression Scale. Nevertheless, 58.1% of patients switched to vortioxetine showed an additional improvement and no clinical worsening was observed in any patients.

All these results probably contributed to the increase of tolerability in sexual dysfunction measured by the SALSEX score, which was observed in patients who retained some sort of SD after switching. This leads us to believe the risk of treatment discontinuation due to SD can be greatly reduced after switching to vortioxetine based on its mechanism of action [41,42].

Finally, the generalized perception of selective serotonin reuptake inhibitors (SSRIs) as the first-line pharmacological treatment of depression needs to move forward to another new antidepressant class due to their adverse effects, such as frequently poorly tolerated sexual dysfunction [43].

### 8.2. Limitations

As this is an observational study, the data obtained may not be representative of the general population, although an attempt to minimize this bias has been made with a calculated sample size of *n* = 124 patients. Due to difficulties in recruitment related to the COVID-19 pandemic, the initial calculated sample size was not reached. Nevertheless, significant differences have been found with this sample of 74 patients at follow-up visit.

Young and elderly people were not included since the study was intended to analyze the adult population. Very few patients over 65 years of age who met the inclusion and exclusion criteria were obtained. Specific studies in these subpopulations would be needed to improve knowledge of and approaches to this clinical issue.

Information was obtained on the decision to switch treatment; no information was obtained on other possible therapeutic alternatives. This information would have to be obtained using different designs in subsequent phases.

## 9. Conclusions

According to our primary objective, sexual functioning significantly improved from baseline to follow-up visit (3 months). Switching to vortioxetine is an effective and reliable strategy to treat patients with poorly tolerated previous antidepressant-related sexual dysfunction and at risk of treatment discontinuation in real-life clinical settings.

The improvement included all components of sexual dysfunction (decreased libido, orgasmic functioning, and arousal difficulties) as well as individual tolerance and risk of treatment discontinuation in both sexes. There seems to be a correlation between the dose and higher sexual dysfunction rates from 20 mg. The switching showed a maintenance of the antidepressant effect in most patients.

## Figures and Tables

**Table 1:** Clinical and sociodemographic characteristics of patients switched to vortioxetine.

	*n* = 74
Age (years)	43.1 ± 12.65
<30, *n* (%)	14 (18.9)
30–40, *n* (%)	21 (28.4)
40–50, *n* (%)	17 (23.0)
>50, *n* (%)	22 (29.7)
Gender (Males), *n* (%)	40 (54.1)
Gender (Females), *n* (%)	34 (45.9)
Duration of treatment (months) (Baseline)	19.53 ± 37.27
Duration of treatment (months) (Follow-up)	3.21 ± 1.17
Dosage (mg/d) (Follow-up)	13.11±4.03
SALSEX Total Score at Baseline	10.32 ± 2.73
SALSEX Total Score at Follow-up	3.78 ± 3.68

**Table 2:** Comparison between sexes in sociodemographic and clinical characteristics of patients switched to Vortioxetine.

Characteristic	Males (*n* = 40)	Females (*n* = 34)	*p*-Value		CI	SE
Age (y.o)	41.28 ± 13.60	45.18 ± 11.26	0.188	t = 1.329	(−1.95, 9.75)	2.93
<30 *n* (%)	11 (27.5)	3 (8.8)				
30–40, *n* (%)	10 (25.0)	11 (32.4)				
40–50, *n* (%)	7 (17.5)	10 (29.4)				
>50, *n* (%)	12 (30.0)	10 (29.4)				
Treatment duration at baseline (months)	14.55 ± 24.02	25.38 ± 48.22	0.129	W = 819.5	-	-
Treatment duration (baseline–follow-up)	3.28 ± 1.41	3.15 ± 0.86	0.900	W = 399	-	-
Vortioxetine dosage (mg/d) (at follow-up))	12.5 ± 4.08	13.82 ± 3.9	0.151	W = 802	-	-
Total score SALSEX—Baseline	9.68 ± 3.16	11.09 ± 1.9	0.021	t = 2.372	(0.22, 2.60)	0.59
Total score SALSEX—Follow-up	2.85 ± 2.8	4.88 ± 4.29	0.021	t = 2.367	(0.31, 3.75)	0.85

**Table 3:** Frequency of sexual dysfunction from baseline to follow-up in Salsex items (%).

	No Dysfunction		Mild		Moderate		Severe	
	Baseline	F-up	Baseline	F-up	Baseline	F-up	Baseline	F-up
Libido decreased	4.1	43.2	10.8	36.5	37.8	12.2	47.3	8.1
Orgasm delayed	1.4	43.2	12.2	40.5	36.5	9.5	50.0	6.8
	Never		Sometimes		Often		Always	
	Baseline	F-up	Baseline	F-up	Baseline	F-up	Baseline	F-up
Anorgasmia	6.8	54.1	24.3	36.5	51.4	5.4	17.6	4.1
Arousal difficulties	9.5	54.1	28.4	32.4	52.7	10.8	9.5	2.7
	No sex dysfunction		Good		Moderate		Poor	
	Baseline	F-up	Baseline	F-up	Baseline	F-up	Baseline	F-up
Acceptability of SD	1.4	34.8	10.8	39.1	47.3	18.8	40.5	7.2

**Table 4:** Comparison between baseline and follow-up in Salsex items. Intensity of SD (%).

	No Sex Dysfunction		Mild		Moderate		Severe	
	Baseline	F-up	Baseline	F-up	Baseline	F-up	Baseline	F-up
Libido decreased								
No problem	21.4	100	14.3	-	35.7	-	28.6	-
Moderate/severe	-	4.5	9.1	27.3	31.8	40.9	59.1	27.3
Orgasm delayed								
No problem	7.1	100	7.1	-	21.4	-	64.3	-
Moderate/severe	-	-	22.7	45.5	13.6	31.8	63.6	22.7
	Never		Sometimes		Often		Always	
	Baseline	F-up	Baseline	F-up	Baseline	F-up	Baseline	F-up
Anorgasmia								
No problem	21.4	100	21.4	-	35.7	-	21.4	-
Moderate/severe	4.5	9.1	27.3	59.1	45.5	18.2	22.7	13.6
Arousal difficulties								
No problem	28.6	100	42.9	-	28.6	-	-	-
Moderate/severe	-	9.1	22.7	45.5	63.6	36.4	13.6	9.1
	No sex dysfunction		Good		Moderate		Poor	
	Baseline	F-up	Baseline	F-up	Baseline	F-up	Baseline	F-up
Acceptability of SD								
No problem	-	100	14.3	-	35.7	-	50.0	-
Poor	-	-	9.1	18.2	50.0	59.1	40.9	22.7

## Data Availability

Data are contained within the article.
